# Candidate biomarkers of PARP inhibitor sensitivity in ovarian cancer beyond the *BRCA* genes

**DOI:** 10.1038/s41416-018-0274-8

**Published:** 2018-10-24

**Authors:** Darren R. Hodgson, Brian A. Dougherty, Zhongwu Lai, Anitra Fielding, Lynda Grinsted, Stuart Spencer, Mark J. O’Connor, Tony W. Ho, Jane D. Robertson, Jerry S. Lanchbury, Kirsten M. Timms, Alexander Gutin, Maria Orr, Helen Jones, Blake Gilks, Chris Womack, Charlie Gourley, Jonathan Ledermann, J. Carl Barrett

**Affiliations:** 1 AstraZeneca, Macclesfield, UK; 2 AstraZeneca, Waltham, MA USA; 3 AstraZeneca, Wilmington, DE USA; 4 Myriad Genetics, Inc., Salt Lake City, UT USA; 5 University of British Columbia, Vancouver, BC Canada; 6 Womack Consulting Ltd, Stamford, UK; 7 Nicola Murray Centre for Ovarian Cancer Research, University of Edinburgh, Edinburgh, UK; 8 UCL Cancer Institute, University College London, London, UK; 9 Present Address: Kesios Therapeutics Ltd, London, UK

**Keywords:** Ovarian cancer, Mutation

## Abstract

**Background:**

Olaparib (Lynparza™) is a PARP inhibitor approved for advanced *BRCA*-mutated (*BRCA*m) ovarian cancer. PARP inhibitors may benefit patients whose tumours are dysfunctional in DNA repair mechanisms unrelated to *BRCA1/2*. We report exploratory analyses, including the long-term outcome of candidate biomarkers of sensitivity to olaparib in *BRCA* wild-type (*BRCA*wt) tumours.

**Methods:**

Tumour samples from an olaparib maintenance monotherapy trial (Study 19, D0810C00019; NCT00753545) were analysed. Analyses included classification of mutations in genes involved in homologous recombination repair (HRR), *BRCA1* promoter methylation status, measurement of BRCA1 protein and Myriad HRD score.

**Results:**

Patients with *BRCA*m tumours gained most benefit from olaparib; a similar treatment benefit was also observed in 21/95 patients whose tumours were *BRCA*wt but had loss-of-function HRR mutations compared to patients with no detectable HRR mutations (58/95). A higher median Myriad MyChoice^®^ HRD score was observed in *BRCA*m and *BRCA*wt tumours with *BRCA1* methylation. Patients without *BRCA*m tumours derived benefit from olaparib treatment vs placebo although to a lesser extent than *BRCA*m patients.

**Conclusions:**

Ovarian cancer patients with tumours harbouring loss-of-function mutations in HRR genes other than *BRCA1*/*2* may constitute a small, molecularly identifiable and clinically relevant population who derive treatment benefit from olaparib similar to patients with *BRCA*m.

## Introduction"

Poly(ADP-ribose) polymerase (PARP) plays a vital role in the repair of single-strand DNA breaks through the base excision repair pathway. PARP inhibitors are thought to become trapped at the sites of single-strand DNA breaks leading to double-strand DNA breaks when DNA replication is attempted.^1^ The double-strand DNA breaks would normally be repaired by the process of homologous recombination repair (HRR), which is a complex process including many proteins, notably *BRCA1* and *BRCA2*.^2^ Tumours with an HRR deficiency, such as those found in *BRCA-*mutated cancers, cannot accurately repair DNA damage when PARP protein function is also disrupted and both the base excision and HRR DNA repair pathways are rendered inoperative. In these tumours, DNA repair by low fidelity repair mechanisms such as non-homologous end joining can cause the accumulation of genomic instability, ultimately resulting in cell death; a concept referred to as synthetic lethality.^3^ Additionally, preclinical data suggest that PARP inhibitors may also benefit patients whose tumours are sensitive to platinum-based chemotherapy and who have an HRR deficiency caused by mutations other than those in the *BRCA1/2* genes.^4,5^ Hence, the clinical and molecular profiles of high-grade serous ovarian cancer (SOC) appeared well matched to PARP inhibitor biology, as this cancer is noted for genome instability thought to be driven by HRR deficiency, repeated and prolonged platinum sensitivity and a high frequency of deficiency in BRCA and other candidate HRR proteins.^6^

Maintenance treatment with the PARP inhibitor olaparib (Lynparza™) in an AstraZeneca-sponsored, randomised, phase II trial of 265 patients with platinum-sensitive relapsed high-grade SOC (Study 19, D0810C00019; NCT00753545) led to a significant increase in progression-free survival (PFS) vs placebo, which was greatest in the subgroup of 136 patients with *BRCA1/2*-mutated tumours (hazard ratio 0.18; 95% confidence interval [CI] 0.10–0.31; *P* *<* 0.001).^7^ This subgroup included patients with inherited and somatic (tumour only) mutations.^7^ Recent analyses strongly suggest that somatic loss-of-function mutations in *BRCA1* and *BRCA2* are an early event in the development of high-grade SOC and that tumours with somatic *BRCA* mutations phenocopy tumours in patients with inherited germline *BRCA* mutations in terms of genetic epidemiology, natural history and response to platinum chemotherapy, and response to olaparib and other PARP inhibitors.^8–13^

Of interest, in Study 19 a significant PFS benefit for olaparib vs placebo was also observed in the subgroup of 118 patients with wild-type *BRCA* tumours, although the treatment benefit was less (PFS hazard ratio 0.54; 95% CI 0.34–0.85; *P* *<* 0.01) than in those patients with *BRCA*-mutated tumours.^7^ A similar trend of broad activity in ovarian cancer with a stronger effect in tumours with *BRCA* mutations has been noted for other PARP inhibitors.^11–13^ Hence, there is considerable interest in understanding the molecular basis of sensitivity to PARP inhibitors in patients whose tumours do not have mutations in the *BRCA* genes and in tumour tests that may aid in the identification of patients who will benefit most from treatment. In particular, an important question to address is whether in *BRCA* wild-type tumours, mutations in other HRR genes account for the benefit observed. To further characterise genetic changes in SOC tumours, we conducted exploratory candidate biomarker analyses on *BRCA* wild-type tumour samples from Study 19 and investigated the possible relationship of HRR deficiencies and clinical benefit.

## Materials and methods

### Study design and population

Study 19 was a phase II, randomised, double-blind, multicentre trial, undertaken at 82 sites in 16 countries. The study design, patient population and statistical analyses have previously been published in detail.^7,14^ In brief, eligible patients were aged 18 years or older and had relapsed SOC (Grade 2 or 3) that was platinum sensitive. Patients entering the study were required to have received two or more previous courses of platinum-based chemotherapy and to have demonstrated an objective response (complete or partial) according to Response Evaluation Criteria in Solid Tumors (RECIST) or Gynecologic Cancer InterGroup criteria. Patients were randomised 1:1 to receive either olaparib 400 mg twice daily (b.i.d.) capsules or matching placebo. Study treatment was continued until progression in the absence of unacceptable toxicity. The primary endpoint was PFS, as determined by RECIST v1.0, and overall survival (OS) was a secondary endpoint.

### Exploratory analyses

To identify patients that benefit from olaparib that do not have *BRCA* mutations, exploratory biomarker analyses were conducted on tumour samples from Study 19 including *BRCA1* promoter methylation, BRCA1 protein expression, HRR gene mutation, and Myriad homologous recombination deficiency (HRD) testing (which includes a tumour test for *BRCA* mutations).

#### BRCA-mutated and BRCA wild-type subgroups in Study 19

Analysis of the *BRCA* mutation status of patients was prespecified in the Study 19 statistical analysis plan. Molecular analyses to define the *BRCA* status of patients in Study 19 were completed retrospectively and were blinded to clinical outcomes.^7^ In brief, the *BRCA*-mutated population comprised patients with a mutation in their tumour and/or blood sample. Germline *BRCA* mutation status was either reported on case report forms after local testing or was established retrospectively using the Integrated BRAC*Analysis*^®^ assay (Myriad Genetics Laboratories, Salt Lake City, UT, USA), with DNA extracted from blood samples obtained prior to randomisation.^15^

The analyses presented here are based on *BRCA* mutation status subgroups defined retrospectively, which were blind to clinical outcomes but were not prespecified in the Study 19 statistical analysis plan.

#### Tumour samples

The provision of an archival tumour sample (blocks or sections) was mandatory for participation in Study 19. Samples received as blocks were made into tumour microarrays with two 0.6 mm cores from each tumour at the University of British Columbia.

#### HRR mutation status

Tumour mutation status in *BRCA1*, *BRCA2* and other key HRR-related genes was established using the same DNA sequencing analysis performed to determine tumour *BRCA* mutation status as previously described.^7^ In brief, DNA was extracted from formalin-fixed, paraffin-embedded (FFPE) archival tumour samples using a cancer gene panel enrichment procedure and deep resequencing performed with Illumina technology. Specifically, analysis was not performed with the commercially available Foundation Focus diagnostic test but with the Foundation Medicine T5 panel (entire coding sequence of 287 cancer-related genes plus select introns from 27 genes and other genetic alterations, deletions and functional rearrangements) at Foundation Medicine (Cambridge, MA, USA).^16^ Tumour analysis was performed on coded tumour samples and results were returned blind to the original Study 19 data set. The classification of variants was based on the American College of Medical Genetics recommendations. Patients with no known *BRCA* mutation and patients with a *BRCA* mutation classed as a variant of unknown significance (VUS) were included in the *BRCA* wild-type group as previously described.^7^

Patients in the *BRCA* wild-type group were further subdivided into three groups: *BRCA* wild-type HRR-mutated, patients whose tumours had a loss-of-function mutation in a high-confidence HRR gene; HRR status unknown, patients with a potential loss-of-function mutation in any gene associated with DNA repair; and *BRCA* and HRR wild type, patients with no potential loss-of-function mutations in any gene involved in DNA repair. The HRR genes interrogated are listed in Supplementary Table 1.

### Myriad MyChoice^®^ HRR deficiency test and *BRCA1* promoter methylation analysis of tumour samples

Tumour samples were analysed as described by Timms et al. (2014).^17^ Next-generation sequencing-based assays were used to generate genome-wide single-nucleotide polymorphism profiles, *BRCA1/2* mutation screening and *BRCA1* promoter methylation data. These analyses separately reported tumour *BRCA* mutation (t*BRCA*m) status, tumour *BRCA1* methylation status and tumour HRD score. An HRD score ≥42 was considered positive. The reported results are based on a research assay performed at Myriad Genetics and not upon the commercially available test.^18^

### Immunohistochemistry for *BRCA1*

FFPE sections of 4 µm thickness were cut and mounted on SuperFrost Plus™ electrostatically charged glass slides and dried overnight at 37 °C in an incubator. Sections were dewaxed in xylene, passed through a graded alcohol series and optimal antigen retrieval obtained using Dako pH 9 antigen retrieval buffer (Dako, Copenhagen, Denmark), heated in the RHS Microwave Histo-processor (Milestone, Italy) to 110 °C with full pressure sustained for 2 min. The pressure vessel was then cooled under running tap water, the lid removed, and the slides washed.

Immunohistochemical staining was performed at room temperature on a Lab Vision 720 Autostainer. Sections were first treated with 3% H_2_O_2_ to quench endogenous peroxidase activity and then blocked with serum-free protein block (Dako). Following blocking, sections were incubated for 60 min with anti-*BRCA1* (Ab-1) mouse monoclonal antibody (MS110) at a concentration of 1 µg/ml (OP92; MerckMillipore, Watford, UK). Visualisation was achieved using Dako REAL™ Envision™, HRP rabbit/mouse (Dako), applied for 30 min, followed by 10 min incubation in 3,3'-diaminobenzidine. Sections were lightly counterstained in Carazzi’s haematoxylin, dehydrated through alcohol grades and mounted under a glass coverslip with Histomount (RA Lamb; Thermo Fisher Scientific, MA, USA).

Staining was negatively controlled by substituting mouse immunoglobulin fraction, diluted to the same concentration as that for the primary antibody. Immunostained slides were evaluated with light microscopy.

### Statistical analysis

The primary endpoint for Study 19 was PFS, which was reported for the full analysis population following a data cutoff (DCO) of 30 June 2010. PFS data were not collected following this DCO. Collection of time to first subsequent therapy or death (TFST), time to second subsequent therapy or death (TSST) and OS data continued, and the interim OS data reported here have a DCO of May 2016.

Analyses of PFS, TFST, TSST and OS use the same methodology; namely a Cox proportional hazards model adjusted for treatment, ethnic descent (Jewish vs non-Jewish), time to progression on penultimate platinum therapy (6–12 vs >12 months), and response to platinum therapy before randomisation (complete response vs partial response). This methodology was used for the primary analyses of the study.^7,14^

Where there were fewer than 15 events formal statistical tests were not performed.

## Results

### Classification of HRR gene mutation status

#### Patients with BRCA wild-type and HRR-mutated tumours or BRCA and HRR wild-type tumours

**Fig. 1:**
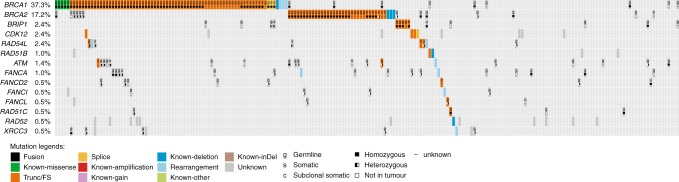
HRR gene mutations that are mutually exclusive to *BRCA1/2*. Each column represents one patient from whom tumours were sequenced (*n* *=* 209)

Archival tumour samples were available for 253/265 (96%) patients and tumour sequencing was completed for 209/265 (79%) patients. Forty-four samples failed due to insufficient tumour cells, DNA quantity, sequencing library quality or sequence read coverage. As previously described,^7^ 111/209 patients were classified as having deleterious or suspected deleterious mutations present in *BRCA1* and/or *BRCA2* in their tumour samples and 98 patients had *BRCA* wild-type tumours; three further patients were known to have single exon indels in blood sample DNA but these were below the detection limit in tumour samples.^7^ Therefore, 95/209 patients (45.5%) were classified as having *BRCA* wild-type tumours. Twenty-one of these patients (22%) had at least one loss-of-function mutation in a candidate HRR gene, of whom 12 received olaparib and 9 received placebo. The specific HRR mutations identified in the 21 tumour samples were: *BRIP1* (*n* = 5), *CDK12* (*n* = 3), *RAD54L* (*n* = 2), *RAD51B* (*n* = 2), *RAD54L* rearr (*n* = 1), *ATM* rearr (*n* = 1), *FANCA* rearr (*n* = 1), *FANCD2* (*n* = 1), *FANCL* rearr (*n* = 1), *FANCL* (*n* = 1), *RAD51C* (*n* = 1), *RAD52* del (*n* = 1), *XRCC3* rearr (*n* = 1) (Fig. 1).

For exploratory purposes, we defined a subset of patients as *BRCA*-mutated or HRR-mutated as a group containing a loss-of-function mutation in at least one pathway gene.

Of the remaining 74 patients, 16 were found to have mutations in genes that are involved more broadly in DNA damage repair (e.g., *MSH2*, *MUTYH)*, or in genes where a postulated role in DNA repair has been the subject of significant controversy (e.g., *EMSY*); these patients were classified as HRR unknown. The remaining 58 patients’ tumours did not have a candidate loss-of-function mutation in a relevant gene. Twenty-five of these patients were randomised to olaparib and 33 to placebo.

### PFS and OS in HRRm and HRRwt patients

**Fig. 2:**
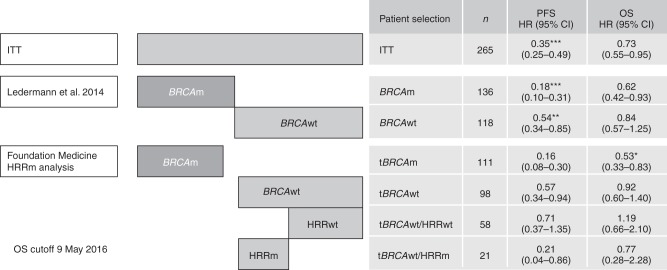
PFS and OS by *BRCA* and HRR status determined at the time of Study 19 and in the exploratory subgroups defined by the Foundation Medicine analysis. *P *<* 0.05, **P *<* 0.01, ***P *<* 0.001. *BRCA*m *BRCA* mutation, *BRCA*wt *BRCA* wild type, CI confidence interval, HR hazard ratio, HRRm homologous recombination repair gene mutation, HRRwt homologous recombination repair genes wild type, *n* number of patients, OS overall survival, PFS progression-free survival, t*BRCA*m tumour *BRCA* mutation, t*BRCA*wt tumour *BRCA* wild type. Three patients who were classified as t*BRCA*wt following Foundation Medicine analyses were found to Have *BRCA*m following germline analysis

PFS was the primary endpoint of Study 19. Patients with a *BRCA* result from tumour testing at Foundation Medicine and *BRCA* wild-type subsets of Study 19 patients had comparable PFS hazard ratios to the overall *BRCA*-mutated (hazard ratio 0.16, 95% CI 0.08–0.3 vs 0.18, 0.10–0.31, respectively) and *BRCA* wild-type populations (hazard ratio 0.57, 0.34–0.94 vs 0.54, 0.34–0.85, respectively) (Fig. 2).^7^

Data suggest that olaparib is associated with a greater PFS benefit in HRR-mutated patients without a *BRCA* mutation (hazard ratio 0.21, 0.04–0.86) than in patients with no detectable *BRCA* or HRR mutation (hazard ratio 0.71, 0.37–1.35) who received olaparib (Fig. 2). Furthermore, all study endpoints showed a consistent trend for a larger treatment effect in HRR-mutated patients and a less positive treatment effect in the patients with no detectable HRR mutations than in the overall *BRCA* wild-type population evaluable for HRR mutation analysis (Supplementary Table 2).

The PFS hazard ratio for the exploratory subgroup of patients with a mutation in a *BRCA* or other HRR gene (*n* = 157) was 0.20, 95% CI 0.12–0.33. It should be noted that PFS in the ITT population was the primary endpoint of the study and hence other analyses are exploratory subgroups of secondary or exploratory endpoints.

### Classification of tumour *BRCA* mutation status, *BRCA1* methylation and HRD score at Myriad

Myriad tumour testing, which comprised *BRCA* mutation testing, *BRCA1* methylation testing and determining HRD score, was performed in 219 tumour samples.

### Myriad tumour *BRCA* testing

Myriad tumour *BRCA* status was reported for 212 patients, of whom 118 (56%) had a *BRCA* mutation. In total, *BRCA* status was determined for tumours from 235 patients using either the Myriad and/or Foundation Medicine analyses. Of the 193 patients for whom tumour *BRCA* status was available at both laboratories, tumour samples from 106 patients were classified as mutated and 84 cases were classed as wt/VUS by both laboratories (VUS samples were predominantly missense variants: impact on gene function is unknown). The concordance of tumour *BRCA* testing between the two laboratories was 98.5% for sequence analysis (Table 1).

**Table 1:** Myriad tumour *BRCA* status compared with Foundation Medicine tumour *BRCA* status

Myriad t*BRCA* status	Foundation Medicine t*BRCA* status			
	*BRCA*m	*BRCA*wt	Missing	Total
*BRCA*m	106	3	9	118
*BRCA*wt	0	84	10	94
Missing	5	11	37	53
Total	111	98	56	265

*BRCAm* *BRCA* mutation, *BRCA*wt *BRCA* wild type, t*BRCA* tumour *BRCA*

**Fig. 3:**
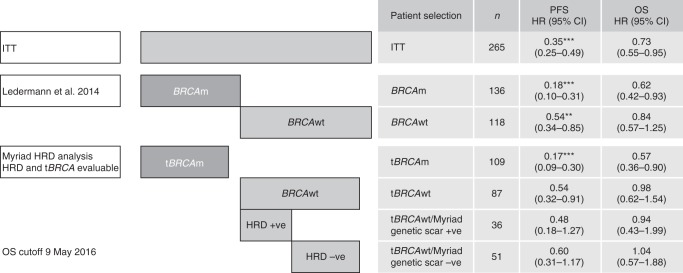
PFS and OS by *BRCA* and HRR status determined at the time of Study 19 and in the exploratory subgroups defined by the Myriad MyChoice HRD score analysis. **P *<* 0.01, ***P *<* 0.001. *BRCA*m *BRCA* mutation, *BRCA*wt *BRCA* wild type, CI confidence interval, HR hazard ratio, HRD homologous recombination deficiency, ITT intention to treat, *n* number of patients, OS overall survival, PFS progression-free survival, t*BRCA*m tumour *BRCA* mutation, t*BRCA*wt tumour *BRCA* wild type

In the tumour samples from three patients, *BRCA* mutations were detected by Myriad testing but not by the Foundation Medicine test. Myriad testing classified the three *BRCA* mutations as a *BRCA1* exon 24 deletion, a *BRCA1* three-exon duplication and a *BRCA2* 32-bp deletion. The efficacy analyses for the Myriad *BRCA-*mutated and wild-type tumours were consistent with the previously published analysis (Fig. 3).^7^

### *BRCA1* methylation

Eighteen of the 212 evaluable patients had *BRCA1-*methylated tumours, of which the tumours from 16 patients were *BRCA* wild type. Four of the 16 *BRCA* wild-type, *BRCA1-*methylated patients were treated with olaparib and 12 with placebo. At the DCO for PFS, 75% of patients in each arm had disease progression.

### Myriad MyChoice homologous recombination deficiency score

Myriad MyChoice HRD scores were determined using an assay that calculates whole-genome tumour loss of heterozygosity, telomeric allelic imbalance and large-scale state transition scores.^17^ The HRD score is the sum of the three scores which have a bimodal distribution and a proposed threshold of ≥42.^17^ Analyses were performed blind to previous *BRCA* mutation data.

HRD scores were determined for 199 patients, of whom 139 (70%) were classified as HRD score high (HRD scores ≥42). Of the 139 tumours with high HRD scores, 101 patients (73%) had a *BRCA* mutation, 36 (26%) had no *BRCA* mutation and *BRCA* analysis failed in two patients (1%). Of the 60 patients (30%) with low HRD scores 8 patients (13%) had a *BRCA* mutation, 51 patients (85%) had no *BRCA* mutation and 1 (2%) had a failed analysis. Overall, 156 patients would be classified as Myriad HRD positive with a *BRCA* mutation in the tumour or a Myriad HRD score ≥42.

### Relationships between HRD score, HRR gene mutations and *BRCA1* methylation status

**Fig. 4:**
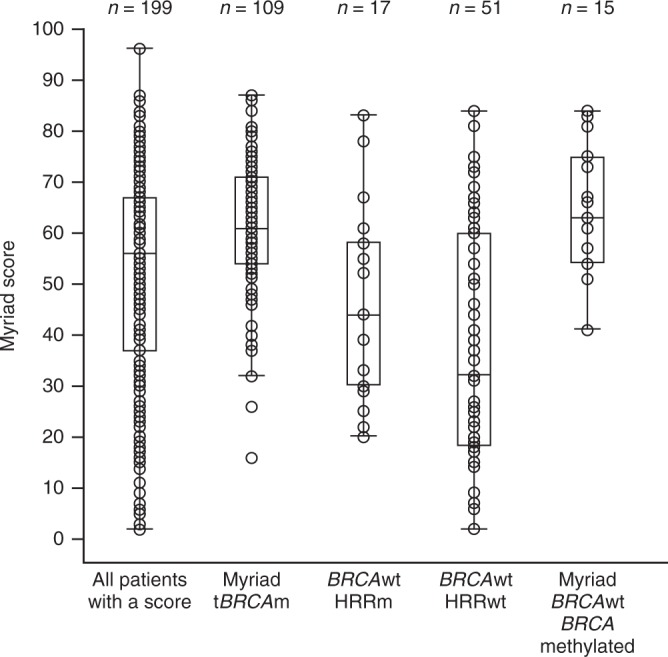
Summary of Myriad MyChoice HRD scores. Patients must be evaluable for both Myriad HRD score and mutation/methylation status to be included. *BRCA*wt *BRCA* wild type, HRD homologous recombination deficiency, HRRm homologous recombination repair gene mutation, HRRwt homologous recombination repair genes wild type, *n* number of patients, t*BRCA*m tumour *BRCA* mutation

*BRCA*-mutated tumours had the highest median HRD score, HRR-mutated tumours had an intermediate median HRD score and tumours without mutations in *BRCA* or other HRR genes had the lowest median HRD score (Fig. 4).

### HRD scores and efficacy

Olaparib is currently approved for use in patients with platinum-sensitive relapsed ovarian cancer (PSROC), regardless of *BRCA* mutation status, in the US (tablet formulation) and in PSROC patients with a *BRCA* mutation in the EU (capsule formulation). We therefore performed exploratory analyses to examine separately the impact of HRD scores within patient groups with *BRCA* wild-type and *BRC*A-mutated tumours defined by Myriad tumour *BRCA* testing. There were only eight patients with *BRCA* mutations and HRD scores <42; this subgroup was not analysed further. When the prespecified threshold of ≥42 was used to define a *BRCA* wild-type HRD high group the PFS hazard ratio (hazard ratio 0.48; 95% CI 0.18–1.27; *P* *=* 0.14; *n* = 36; 20 olaparib, 16 placebo) was numerically superior to the PFS hazard ratio for the *BRCA* wild-type HRD low group (hazard ratio 0.60; 95% CI 0.31–1.17, *P* *=* 0.13; *n* = 51; 25 olaparib, 26 placebo) but was very similar to that for the overall *BRC*A wild-type population evaluable for HRD scores (hazard ratio 0.54; 95% CI 0.32–0.91; *P* *=* 0.02; *n* = 87) (Fig. 3). Further exploratory analyses did not show a trend for an improved hazard ratio for PFS with higher Myriad HRD scores in either the *BRCA* wild-type or mutated populations when the HRD cut-off was varied (data not shown). However, the relevant extremes of the HRD score distribution were only populated by small numbers of patients such that very few tumours with *BRCA* mutations had scores below 30 (*n* = 1) and conversely few tumours without *BRCA* mutations had scores greater than 50 (*n* = 30). Similar marginal differences in the hazard ratios observed for PFS between the *BRCA* wild-type HRD high group and the *BRCA* wild-type HRD low group were broadly observed for TFST, TSST and OS (Supplementary Table 3).

The PFS hazard ratio for the exploratory subgroup of patients with HRD-positive status (*n* = 156) was 0.24, 95% CI 0.15–0.39. It should be noted that PFS in the ITT population was the primary endpoint of the study and hence other analyses are exploratory subgroups of secondary or exploratory endpoints.

A recent study suggested patients with tumours with HRD scores <33 are less likely to benefit from platinum chemotherapy.^19^ In Study 19 this patient population, *BRCA*wt/HRD<33, had a worse PFS result (PFS hazard ratio 0.71, 95% CI 0.34–1.46, *n* = 41; 21 olaparib, 20 placebo) than the HRD-negative group (*BRCA*wt, HRD score <42) or the *BRCA*-mutated and/or HRD ≥33 group (PFS hazard ratio 0.25, 95% CI 0.16–0.38, *n* = 185, 97 olaparib, 88 placebo).

### Markers associated with long-term treatment

In Study 19 there were 16 patients treated for 6 years or longer, of whom one was on placebo and had a germline mutation in *BRCA1*. Of the 15 patients on olaparib for more than 6 years, 8 were originally reported as having *BRCA* mutations.^7^ One further patient was found to have somatic mutations in both *BRCA1* and *BRCA2* on Myriad blood and tumour testing (no Foundation Medicine tumour testing data were available); hence, 9/15 patients are known to have a *BRCA* mutation (5 with *BRCA2*, 3 with *BRCA1* and 1 with both) in their tumour or blood sample, with 3/9 being a somatic mutation. This enrichment for *BRCA2* mutation and presence of somatic cases in long-term responders has been noted previously.^20^ One of the remaining six patients was not evaluable for t*BRCA* status at either Myriad or Foundation Medicine, hence 5/15 patients treated with olaparib for more than 6 years had no detectable *BRCA* mutation. Three out of five patients had Myriad HRD scores available (2x HRD positive and 1x HRD negative) and 5/5 had HRRm status available (1x HRRm, 2x HRR uncertain, 2x HRRwt). Of the two HRRwt tumours, one was Myriad HRD negative (Myriad HRD score 15) and the other was Myriad HRD status unknown. Therefore, patients without *BRCA*-mutated tumours and whose tumours tested negative for additional candidate biomarkers (HRRwt or *BRCA*wt HRD negative) were found among the patients who remained on olaparib for over 6 years.

### Immunohistochemistry for *BRCA1*

Staining was performed on duplicate cores on the tissue microarrays generated from the 145 patients submitting a tumour block (Supplementary Table 4). A trend for less staining in *BRCA1*-mutated cases was observed (Supplementary Table 5). However, there was no indication of a prognostic or predictive effect on PFS of BRCA1 protein levels determined by immunohistochemical staining using Cox regression analysis.

## Discussion

Elegant preclinical experiments led to the concept of synthetic lethality, which suggested that tumours without functional BRCA1 or BRCA2 protein would be more susceptible to PARP inhibitors compared with non-tumour tissues in the same patients.^21^ In this model, PARP inhibition leads to an accumulation of unrepaired single-strand breaks in DNA, which are converted to double-strand breaks when the tumour cell attempts DNA replication in the S-phase. The resulting double-strand breaks are then repaired in non-tumour cells that are proficient in HRR but accumulate and lead to mitotic catastrophe in tumour cells that are HRR deficient. *BRCA1* and *BRCA2* play important roles in homologous recombination and, in germline carriers of mutations, loss of the remaining un-mutated copy of the gene and concomitant loss of function is thought to occur early in the development of ovarian and breast cancer.^22^ Hence, early clinical studies with the PARP inhibitor olaparib focused on tumours in germline-mutated *BRCA* carriers and confirmed activity in patients with breast and ovarian cancer.^23,24^ Preclinically, deficiencies in other proteins involved in homologous recombination conferred sensitivity to PARP inhibition, albeit with a lesser impact than *BRCA* deficiency^5^ and high-grade SOC was initially thought to represent a particularly promising tumour type for PARP inhibitor therapy because, in addition to a high frequency of *BRCA* mutations, it is characterised by large-scale genomic instability and repeated, durable platinum sensitivity (considered likely to be hallmarks of HRR deficiency). In support of this theory, a non-randomised study of olaparib monotherapy in ovarian cancer subsequently found response rates of 41% and 24% in the germline *BRCA*-mutated and non-mutated carrier patient populations, respectively.^25^ Therefore, it was expected that the non-*BRCA-*mutated carrier high-grade SOC patient population included a significant number of patients whose tumours could be susceptible to PARP inhibition and Study 19 was a placebo-controlled study in the maintenance setting designed to test this hypothesis.^7^ Study 19 was a positive study overall and in a preplanned subgroup analysis the magnitude of PFS effect was greater in patients who had a *BRCA* mutation than those without.^7^ Tumour testing in Study 19 successfully identified the vast majority of germline *BRCA*-mutated cases and identified a further subset of patients with somatic or tumour only mutations.^7^ Subsequent exploratory analyses suggested that tumours with somatic *BRCA* mutations phenocopy those from germline carriers, both in terms of tumour genetics and in clinical benefit on olaparib^10^ and a clinical trial is under way to confirm this observation.^26^ Hence, *BRCA* mutation testing of tumour samples is the clearest indicator of tumour responsiveness to olaparib treatment in current clinical practice. Importantly, the data presented here confirm a very high level of consistency in research testing for tumour *BRCA* mutations in two independent laboratories and therefore confirms our previous observation that tumour testing accurately detects germline mutations. The number of mutations not captured by the Foundation Medicine assay was relatively small (*n* = 3, all indels). Nonetheless, it is important that all tumour testing laboratories further develop their methodologies to accurately detect rare variants to allow tumour testing to detect all clinically relevant mutations.

We have shown that Study 19 contained a readily identifiable, biologically coherent subpopulation of patients with inherited or acquired mutations in *BRCA1* or *BRCA2* who gain a PFS benefit from olaparib treatment. It is important that we determine whether there is a molecularly identifiable subgroup of patients with *BRCA* wild-type tumours that is differentiated from patients solely selected on available clinical parameters of platinum sensitivity and high-grade serous histology used in Study 19. Furthermore, if such a subgroup exists it will be important to determine whether the differential in benefit is clinically meaningful. Several candidate molecular measures have been suggested to fulfil this role including a continuous measure of genomic instability or HRR deficiency ‘scar’, *BRCA1* methylation and loss-of-function mutations in other homologous recombination pathway genes. The supporting data behind the candidate biomarkers investigated are summarised in Table 2.

**Table 2:** Summary of data to support investigation of candidate biomarkers

Candidate biomarker	Summary of evidence base	References
*BRCA1* methylation	Multiple independent reports in ovarian cancer suggesting (i) mutual exclusivity with *BRCA1* mutations (ii) coincidence with *BRCA1* loss of heterozygosity. However, no reported *BRCA*m-like link to good prognosis and platinum sensitivity	^37, 38^
BRCA1 protein	Low protein levels linked to increased duration of survival in platinum-treated ovarian cancer. However, some difficulties in reproducing data	^39– 41^
HRR gene mutations	Preclinically linked to PARP inhibitor sensitivity. Mechanistically analogous to *BRCA*m and linked to prolonged survival and platinum sensitivity in ovarian cancer. However, difficult to gauge impact of individual genes due to low prevalence and some evidence of non-mutual exclusivity	^5, 8^
HRD score	Sensitive for *BRCA*m and *BRCA1* methylation, relatively conserved between primary and metastatic lesions. Prognostic for and linked to higher platinum response rate. Evidence from ovarian cancer trials: shorter progression-free survival in patients with HRD-positive tumours without *BRCA* mutations vs those with *BRCA* mutations. Longer progression-free survival in patients with HRD-positive vs HRD-negative tumours; however, may serve as a genomic scar that is irreversible and unreflective of current tumour phenotype and, unlike *BRCA*m, was not predictive for platinum vs taxane in the TNT trial in breast cancer	^11, 12, 17, 42, 43^

*BRCA*m *BRCA* mutation, *HRD* homologous recombination deficiency, *HRR* homologous recombination repair

Here, we report that the ovarian *BRCA* wild-type patient population may include a majority of patients whose tumours lack DNA repair gene loss-of-function mutations and who gain less benefit from PARP olaparib treatment than patients with candidate loss-of-function mutations in genes involved in HRR.

First, we attempted to identify patients with loss-of-function mutations in candidate HRR genes in their tumours. Clearly this approach is limited by the genes sequenced by the Foundation Medicine panel and by the relative absence of relevant preclinical and clinical data. We therefore chose a pragmatic approach to include all genes with a DNA repair pathway role and some supportive preclinical data. Subsequently, we defined a higher confidence patient population by differentiating between candidate genes in which the primary role is related to mismatch repair or where, despite a purported role in double-strand DNA repair, either the genetic mechanism is distinct and/or the phenotypic impact on platinum sensitivity and prognosis in ovarian cancer is directionally opposed to that of *BRCA* mutations (i.e., *EMSY* amplification and *PTEN* loss).^8^

We identified 37 patients with mutations in any gene involved in DNA repair, of these patients 21 had mutations in the (relatively) higher confidence gene set. Genes involved in more than one patient were *BRIP1* (*n* = 5), *CDK12* (*n* = 3), *RAD54L* (*n* = 2) and *RAD51B* (*n* = 2). *BRIP1* (BRCA1 interacting protein C-terminal helicase 1) is a protein that works with BRCA1 to repair damaged DNA and the inheritance of two mutated copies of *BRIP1* causes Fanconi anaemia. Women who inherit one mutated copy of *BRIP1* are likely to have an increased risk of ovarian cancer; however, the implication of this for breast cancer risk is inconclusive.^27–29^

*CDK12* is the least validated gene included in our HRR gene list. It is biallelically inactivated in ovarian cancer; hypothesis-directed experiments have found that mutations or depletion are associated with sensitivity to radiation, platinum or PARP inhibitor treatment and it was also highlighted as a candidate gene in a hypothesis-free synthetic lethal screen approach.^30,31^ However, mechanistically it has been proposed to have an indirect effect on DNA repair via transcriptional regulation^32^ and it was recently suggested to be associated with a different pattern of genome instability than that seen in patients with *BRCA* mutations or *BRCA1* methylation.^33^

The number of patients with mutations in any HRR gene (other than *BRCA1* or *BRCA2*) in Study 19 is too small to derive definitive conclusions. Furthermore, because of the low prevalence of mutations in any one gene a similarly sized trial could easily result in a different profile of mutated genes. However, it was possible to define a subpopulation of 58 patients without a candidate loss-of-function mutation in any measured relevant HRR gene. While it is acknowledged that mutations or alterations in other unmeasured genes may not have been detected in these 58 patients, it is notable that for all efficacy measures there was marked attenuation of the treatment benefit from olaparib. Our data are consistent with those of another study,^8^ suggesting that tumours with mutations in HRR genes may be associated with increased platinum sensitivity and prolonged survival.

While these data are encouraging, significant challenges remain, due to the differing biological role of each gene and the low prevalence of mutations in any one gene. Hence, determining utility for clinical decision-making suffers from the analogous issues facing genetic teams with low prevalence/penetrance inherited gene mutations, although the risk–benefit profile for the treatment decisions in a cancer patient may be arguably different to that for prophylactic surgery. Further data are needed to confirm this observation and there is a need for the clinical, research and regulatory community to understand what evidence base can be required for individual or ‘buckets’ of low prevalence genes with high biological plausibility. Exploratory analyses of the recently reported ARIEL2, ARIEL3 and NOVA trials could significantly add to the present evidence base available.^11–13^

The ability to avoid these issues for causal genetic changes and use an HRD score or genomic read-out of the effects of HRD, regardless of causal mechanism, is inherently attractive in theory. In Study 19 there were only 36 patients with *BRCA* wild-type tumours with a high Myriad HRD score. In agreement with data presented from a preliminary analysis of the NOVA trial we note that HRR-mutated patients tend to have higher Myriad HRD scores in Study 19 but that *BRIP* mutations appear to have a weak correlation such that, in both Study 19 and the preliminary NOVA data, only half of *BRIP*-mutated tumours were HRD score positive. In Study 19 only half of all the HRD score evaluable HRR-mutated patients were HRD positive (scores ≥42), whereas 14 of the 15 patients with *BRCA1* methylation who were evaluable for HRD score were HRD positive. *BRCA1*-methylated cases accounted for at least 39% of the *BRCA* wild-type HRD-positive population in Study 19. An exploratory analysis of the 36 *BRCA* wild-type Myriad HRD-positive cases suggested that these patients were only marginally more likely to benefit from olaparib than selection based on study entrance criteria (response to platinum and high-grade serous histology); it may be that this is largely because of an inability of *BRCA1* methylation to phenocopy *BRCA1* mutation in terms of olaparib sensitivity/ease of reversibility as has also been reported for platinum sensitivity.^8^ The *BRCA* wild-type HRD scores ≥42 analysis reported here is an exploratory analysis of a small number of patients (*n* = 36) and aligns with the recently reported data for the NOVA and ARIEL3 trials which used the Myriad HRD (42) score and Foundation Medicine LOH score, respectively.^12,13^ In further exploratory analysis of Study 19, we show that relaxing the HRD threshold to a value of 33 differentiates a potential population receiving enhanced benefit compared to patients below the new threshold (hazard ratio 0.71 in HRD score <33 vs hazard ratio = 0.25 in HRD score ≥33). Particularly in a setting where the majority of patients appear to derive some level of benefit from PARP inhibitors, considering a biomarker that aims to exclude those patients least likely to benefit, rather than identifying those that are most likely to benefit, might be an attractive strategy. Compared to the HRD threshold of 42, this modified HRD scar approach, using a threshold <33, may have the potential to better identify a patient population that would derive least benefit from PARP inhibitors, but requires further study.

Importantly, although we observed an enrichment for *BRCA2* mutations in the long-term responders, neither an HRRm or HRD approach was able to exclude patients who remained on treatment for 6 years or more. It is likely that additional mechanisms, such as those relating to the immune system, are important in determining which patients may receive long-term benefit from olaparib and potentially also from chemotherapy.^34,35^

## Conclusions

In summary, the analyses reported here demonstrate that next-generation sequence-based tumour testing for *BRCA* mutations in clinically available FFPE samples can be highly reproducible and may identify a subpopulation of patients with the greatest PFS benefit from olaparib. A measure of HRD or genomic instability (Myriad MyChoice HRD) identified most patients with *BRCA* mutations and nearly all patients with *BRCA1* methylation; however, only ~50% of a small number of patients with mutations in other candidate HRR genes were identified as HRR deficient. In underpowered exploratory analyses (i) patients with *BRCA* wild-type HRD-positive tumours did not achieve as high a treatment benefit from olaparib as patients with *BRCA*-mutated tumours and (ii) patients with tumours without mutations in candidate HRR genes, which comprised the majority of the *BRCA* wild-type subpopulation, received the least benefit from olaparib treatment, an observation which may be particularly important in the maintenance setting in the absence of any acute indicator of tumour response to olaparib treatment.

Further clinical studies examining all candidate tumour DNA-based measures of cause and effect deficiencies in HRR pathways are required to confirm that these additional measurements can provide clinically relevant information and treatment benefits for patients with *BRCA* wild-type high-grade SOCs.

### Availability of data and materials

AstraZeneca’s policy on clinical trial data, results and other information from or regarding AstraZeneca-sponsored clinical trials are described in full in the AstraZeneca website: https://astrazenecagrouptrials.pharmacm.com/ST/Submission/Search

## Electronic supplementary material

Supplementary material
