# Supplementary material for: Candidate biomarkers of PARP inhibitor sensitivity in ovarian cancer beyond the BRCA genes
Source: Br J Cancer. 2018 Oct 24;119(11):1401–9. doi: 10.1038/s41416-018-0274-8 (PMC6265286; doi:10.1038/s41416-018-0274-8)
Supplement: Supplementary file 1 — Supplementary material [file 41416_2018_274_MOESM1_ESM.docx]

## Supplementary material

# Candidate biomarkers of PARP inhibitor sensitivity in ovarian cancer beyond the *BRCA* genes

Darren R Hodgson*^1^, Brian A Dougherty^2^, Zhongwu Lai^2^, Anitra Fielding,^1^ Lynda Grinsted^1^, Stuart Spencer^1^, Mark J O’Connor^1^, Tony W Ho^3^, Jane D Robertson^1†^, Jerry S Lanchbury^4^, Kirsten M Timms^4^, Alexander Gutin^4^, Maria Orr^1^, Helen Jones^1^, Blake Gilks^5^, Chris Womack^6^, Charlie Gourley^7^, Jonathan Ledermann^8^ and J Carl Barrett^2^

Supplementary Table 1. A list of genes profiled on the Foundation Medicine T5 panel diagnostic test that are considered to be involved in homologous recombination repair (HRR)

| ATM | CDK12 | FANCG | PALB2 | RAD51C |
| --- | --- | --- | --- | --- |
| ATR | CHEK1 | FANCI | PARP1 | RAD51D (RAD51L3) |
| ATRX | CHEK2 | FANCL | PARP2 | RAD52 |
| BACH1 | CUL4A | FANCM | PARP3 | RAD54L |
| BAP1 | EMSY (c11orf30) | MLH1 | PARP4 | RPA1 |
| BARD1 | FANCA | MRE11A | PMS2 | TIPARP |
| BLM | FANCC | MSH2 | PRKDC | XRCC3 |
| BRCA1 | FANCD2 | MSH6 | RAD50 |  |
| BRCA2 | FANCE | MUTYH | RAD51 |  |
| BRIP1 | FANCF | NBN | RAD51B (RAD51L1) |  |

Supplementary Table 2. Efficacy analyses of PFS, TFST, TSST and OS in 21 HRRm BRCAwt and 58 BRCAwt HRRwt patients determined by Foundation Medicine analysis

| *BRCAwt* HRRm | | | | |
| --- | --- | --- | --- | --- |
|  | Events:Patients (%) | Hazard ratio | 95% CI | Two-sided *P* value |
| PFS | Olaparib 400 mg bid: 7:12 (58.3)  Placebo: 6:9 (66.7) | 0.21 | 0.04–0.86 | *P* = 0.03 |
| TFST | Olaparib 400 mg bid: 11:12 (91.7)  Placebo: 9:9 (100) | 0.54 | 0.18–1.60 | *P*=0.26 |
| TSST | Olaparib 400 mg bid: 11:12 (91.7)  Placebo: 9:9 (100) | 0.58 | 0.19–1.75 | *P*=0.33 |
| OS | Olaparib 400 mg bid: 11:12 (91.7)  Placebo: 8:9 (88.9) | 0.77 | 0.28–2.28 | *P*=0.63 |
| *BRCAwt* HRRwt | | | | |
|  | Events:Patients (%) | Hazard ratio | 95% CI | Two‑sided *P* value |
| PFS | Olaparib 400 mg bid: 17:25 (68.0)  Placebo: 23:33 (70) | 0.71 | 0.37–1.35 | *P*= 0.30 |
| TFST | Olaparib 400 mg bid: 21:25 (84.0)  Placebo: 32:33 (97) | 0.65 | 0.36–1.13 | *P*= 0.13 |
| TSST | Olaparib 400 mg bid: 21:25 (84.0)  Placebo: 32:33 (97) | 0.93 | 0.52–1.64 | *P*= 0.81 |
| OS | Olaparib 400 mg bid: 21:25 (84.0)  Placebo: 32:33 (97) | 1.19 | 0.66–2.1 | *P*= 0.55 |

bid, twice daily; CI, confidence interval; OS, overall survival; PFS, progression-free survival; TFST, time to first subsequent therapy; TSST, time to second subsequent therapy

Supplementary Table 3. Efficacy analyses of PFS, TFST, TSST and OS in 36 BRCAwt HRD positive and 51 BRCAwt HRD negative patients defined by the Myriad MyChoice HRD score analysis

| *BRCAwt* HRD positive | | | | |
| --- | --- | --- | --- | --- |
|  | Events:Patients (%) | Hazard ratio | 95% CI | Two‑sided *P* value |
| PFS | Olaparib 400 mg bid: 8:16 (50)  Placebo: 11:20 (55) | 0.48 | 0.18–1.27 | *P*= 0.14 |
| TFST | Olaparib 400 mg bid: 12:16 (75.0)  Placebo: 20:20 (100) | 0.61 | 0.27–1.31 | *P*= 0.21 |
| TSST | Olaparib 400 mg bid: 12: 16 (75)  Placebo: 20:20 (100) | 1.04 | 0.46–2.23 | *P*= 0.93 |
| OS | Olaparib 400 mg bid: 12:16 (75)  Placebo: 19:20 (95) | 0.94 | 0.43–1.99 | *P*= 0.87 |
| BRCAwt HRD negative | | | | |
|  | Events:Patients (%) | Hazard ratio | 95% CI | Two‑sided  *P* value |
| PFS | Olaparib 400 mg bid: 18:26 (69.2)  Placebo: 21:25 (84.0) | 0.60 | 0.31–1.17 | *P*= 0.13 |
| TFST | Olaparib 400 mg bid: 22:26 (84.6)  Placebo: 25:25 (100) | 0.45 | 0.24–0.82 | *P*= 0.01 |
| TSST | Olaparib 400 mg bid: 22:26 (84.6)  Placebo: 25:25 (100) | 0.60 | 0.32–1.09 | *P*= 0.10 |
| OS | Olaparib 400 mg bid: 22:26 (84.6)  Placebo: 25:25 (100) | 1.04 | 0.57–1.88 | *P*= 0.90 |

bid, twice daily; CI, confidence interval; OS, overall survival; PFS, progression-free survival; TFST, time to first subsequent therapy; TSST, time to second subsequent therapy

Supplementary Table 4. Concordance of BRCA1 staining intensity for duplicate cores

|  | Sample 2 | | | | | | | |
| --- | --- | --- | --- | --- | --- | --- | --- | --- |
| Sample 1 | 0 | 1 | 2 | 3 | NT | FC | NC | Total |
| 0 | 61 | 12 | 0 | 0 | 7 | 0 | 0 | 80 |
| 1 | 10 | 28 | 4 | 1 | 2 | 0 | 0 | 45 |
| 2 | 0 | 2 | 4 | 0 | 0 | 1 | 0 | 7 |
| NC | 0 | 0 | 0 | 0 | 0 | 1 | 1 | 2 |
| NT | 2 | 1 | 1 | 0 | 7 | 0 | 0 | 11 |
| Total | 73 | 43 | 9 | 1 | 16 | 2 | 1 | 145 |

*BRCA*m immunohistochemical staining score was defined as: 1, weak; 2, moderate; 3, strong.

FC, fold change; NC, no core; NT, no tumour

Supplementary Table 5. *BRCA1* immunohistochemical staining score concordance with *BRCAm* status defined in Study 19

| *BRCA*m status determined in Study 19 | *BRCA*1 immunohistochemical staining score* | | | |
| --- | --- | --- | --- | --- |
|  | 0 | 1 | 2 | Missing |
| BRCA1 | 40 | 9 | 0 | 43 |
| BRCA2 | 12 | 10 | 1 | 20 |
| BRCA1/2 | 0 | 0 | 0 | 1 |
| *BRCA*wt/VUS | 36 | 19 | 5 | 58 |
| Missing | 4 | 0 | 0 | 7 |

*BRCA*m immunohistochemical staining score was defined as: 1, weak; 2, moderate; 3, strong.

*Data derived from Supplementary Table 2: If either score was 0 then the combined score was 0, otherwise if either score was 1 then the combined score was 1, otherwise if either score was 2 then the combined score was 2, if either score was 3 then the combined score was 3
